# Neuroprotective Potential of Peroxisome Proliferator Activated Receptor-****α**** Agonist in Cognitive Impairment in Parkinson's Disease: Behavioral, Biochemical, and PBPK Profile

**DOI:** 10.1155/2014/753587

**Published:** 2014-02-19

**Authors:** Dedeepya Uppalapati, Nihar R. Das, Rahul P. Gangwal, Mangesh V. Damre, Abhay T. Sangamwar, Shyam S. Sharma

**Affiliations:** ^1^Molecular Neuropharmacology Laboratory, Department of Pharmacology and Toxicology, National Institute of Pharmaceutical Education and Research (NIPER), Sector 67, SAS Nagar, Punjab 160062, India; ^2^Department of Pharmacoinformatics, National Institute of Pharmaceutical Education and Research (NIPER), Sector 67, SAS Nagar, Punjab 160062, India

## Abstract

Parkinson's disease (PD) is a common neurodegenerative disorder affecting 1% of the population by the age of 65 years and 4-5% of the population by the age of 85 years. PD affects functional capabilities of the patient by producing motor symptoms and nonmotor symptoms. Apart from this, it is also associated with a higher risk of cognitive impairment that may lead to memory loss, confusion, and decreased attention span. In this study, we have investigated the effect of fenofibrate, a PPAR-**α** agonist in cognitive impairment model in PD. Bilateral intranigral administration of 1-methyl-4-phenyl-1,2,3,6-tetrahydropyridine (MPTP) (100 µg/1 µL/side) produced significant cognitive dysfunctions. Fenofibrate treatment at 10, 30, and 100 mg/kg for twenty-five days was found to be neuroprotective and improved cognitive impairment in MPTP-induced PD model as evident from behavioral, biochemical (MDA, GSH, TNF-**α**, and IL-6), immunohistochemistry (TH), and DNA fragmentation (TUNEL positive cells) studies. Further, physiologically based pharmacokinetic (PBPK) modeling study was performed using GastroPlus to characterize the kinetics of fenofibric acid in the brain. A good agreement was found between pharmacokinetic parameters obtained from the actual and simulated plasma concentration-time profiles of fenofibric acid. Results of this study suggest that PPAR-**α** agonist (fenofibrate) is neuroprotective in PD-induced cognitive impairment.

## 1. Introduction

Parkinson's disease (PD) is the chronic, age-related neurodegenerative disorder of the central nervous system characterised by progressive loss of dopaminergic neurons in the substantia nigra pars compacta (SNpc) leading to dopamine deficiency in the striatum. Though the cause of nigral cell death and underlying mechanism has not been clearly elucidated yet [1], many reports have suggested that increased oxidative stress [2], inflammation [3], mitochondrial dysfunction [4], excitotoxicity [5], and proteasomal dysfunction [6] play key role in initiating and mediating cell death. PD causes both motor symptoms which mainly include tremor at rest, rigidity, akinesia, and postural instability and nonmotor symptoms like cognitive impairment, autonomic dysfunction, and sensory and sleep disturbances [7]. Nearly 40% of the PD patients are affected with cognitive impairment and dementia [8]. The risk of incidence of dementia augments with the older age is up to 6 times higher in PD patients when compared to the healthy people [9, 10].

Newer strategies are required for neuroprotection and amelioration of cognitive dysfunction in PD. Though it is well known that peroxisome proliferator activated receptor (PPAR) agonists protect against oxidative damage, inflammation, apoptosis in periphery, recent literature have described the neuroprotective role of PPAR agonists in CNS disorders [11]. PPARs are a group of nuclear receptor transducer proteins that functions as ligand-regulated transcription factors regulating the expression of genes [12]. Neuroprotective effects of PPARs have been described in various neurodegenerative disorders like Alzheimer's disease [13], stroke [14], Huntington's disease [15], and multiple sclerosis [16]. PPAR agonists have also shown to be effective in several *in vitro* and *in vivo* models of PD. PPAR-*γ* agonists pioglitazone [17] and rosiglitazone [18] were shown to exert protective effects in a mouse model of PD. Recently, neuroprotective effects of PPAR-*δ* agonist GW0742 were described, whereas PPAR-*δ* antagonist GSK0660 enhanced the detrimental effects of MPP+ on cell viability [19]. Two PPAR-*α* agonists were investigated for neuroprotective effect in MPTP mouse model of PD and interestingly fenofibrate showed neuroprotective effect whereas bezafibrate did not [20].

Although 40% of the PD patients suffer from cognitive impairment and dementia, still there is no satisfactory drug for cognitive impairment associated with PD. Earlier we have demonstrated that PPAR-*γ* agonist pioglitazone significantly improved cognitive impairment in PD [21]. PPAR-*α* agonist fenofibrate has shown to be effective in cognitive impairment in various disease conditions. However, the effect of fenofibrate on cognitive impairment in PD has not been reported yet. In the present study, the effect of fenofibrate in cognitive impairment has been investigated in MPTP-induced PD in rat model by assessing various behavioral and biochemical parameters, immunohistochemistry and DNA fragmentation studies. It was further correlated by physiologically based pharmacokinetic (PBPK) modeling study for fenofibric acid.

## 2. Materials and Methods

### 2.1. Animals

Male Sprague Dawley rats (280–320 g) were obtained from Central Animal Facility (CAF), National Institute of Pharmaceutical Education & Research (NIPER), S.A.S. Nagar, Punjab, India. They were provided with standard pellet diet and water ad libitum. They were kept at room temperature 22 ± 2°C, humidity 55 ± 5%, and 12 h light/dark cycle. All the experimental protocols were approved by the Institutional Animal Ethics Committee of NIPER.

### 2.2. Bilateral Intranigral Administration of MPTP

Experimental MPTP models have been developed to mimic human PD and serve as an indispensable tool in PD. Intranigral administration of MPTP was carried out as described by Da Cunha et al. with slight modification [22]. Briefly, rats were given atropine sulphate (0.4 mg/kg, i.p.) as preanesthetic medication and were anesthetized with sodium thiopental (50 mg/kg, i.p.). MPTP.HCl (100 *μ*g/1 *μ*L of saline/side) was bilaterally infused using 5 *μ*L microlitre syringe at the following coordinates of SNc: anteroposterior (AP): −5.0 mm from bregma; mediolateral (ML): ±2.1 mm from midline; dorsoventral (DV): −7.7 mm from skull. Rats in sham-operated group were subjected to the same procedure with the infusion of 1 *μ*L of saline instead of MPTP bilaterally into the SNc.

### 2.3. Treatment Schedule

The rats were randomly divided into the following groups: Sham, MPTP, MPTP + vehicle (0.5% carboxymethyl cellulose), MPTP + fenofibrate (10 mg/kg), MPTP + fenofibrate (30 mg/kg), and MPTP + fenofibrate (100 mg/kg). Fenofibrate was suspended in 0.5% carboxymethyl cellulose. Fenofibrate was administered orally for 5 days before (i.e. D-5 to D0) MPTP injection (considered as D0) and continued for next twenty-five days. Each group consisted of eight to twelve animals. After behavioral studies, animals were sacrificed and used for biochemical and histological studies.

### 2.4. Behavioral Parameters 

#### 2.4.1. Passive Avoidance Test

This test was performed on 19th and 20th day after the bilateral intranigral administration of MPTP. The apparatus (Columbus Instruments, USA) used for the study consisted of two compartments, an illuminated light chamber and a dark chamber separated by an automatically operated sliding door. The rat got an initial habituation for a period of 60 s in the light chamber after which sliding door opened and it entered into the dark chamber where it got a mild foot shock of 0.6 mA for 6 s through the grid floor. The time taken by the rat to step into the dark compartment was recorded as initial trial latency (ITL). The rats which did not enter into the dark chamber within the cut-off time of 60 s were not considered for further experiments. After 24 h, retention trial was performed and latency to step into the dark compartment was recorded as retention trial latency (RTL) to a maximum of 300 s [21, 23, 24].

#### 2.4.2. Morris Water Maze Test

This test was conducted 21 days after MPTP administration. Water maze consisted of a large circular pool divided into four imaginary quadrants. A submerged platform (10 cm × 10 cm) was placed 2 cm below the surface of water in the center of one of the quadrants. The position of the submerged (escape) platform was cued from different object locations in the room. In our study platform position was kept constant throughout the trial while the animal position was changed in each trial. To escape the swimming rat climbed this platform. The rats were given four acquisition trials per day for 5 days with an intertrial interval of maximum of 360 s. Rats were allowed to locate the platform for maximum of 120 s during acquisition period. Those who failed to reach the platform were drifted towards it and then permitted to stay on it for 20 s. On 6th day of the test, retention trial was conducted in which the platform was removed and the rats were given 120 s to explore the previous location of the platform. During the trials, the pool was videotaped and the escape latency (time taken to find the hidden platform) and the number of entries to the platform zone were recorded by Any-Maze software (Stoelting, USA) [21, 25].

### 2.5. Biochemical Parameters

After behavioral experiment, the animals were sacrificed by decapitation and the brain was taken out. Mid-brain tissue was then homogenized in 0.1 M phosphate buffer (pH 7.4, 5 mL/g of tissue) using Polytron homogenizer and homogenate was used for estimation of oxidative stress parameters.

#### 2.5.1. Malondialdehyde (MDA) Estimation

Estimation of MDA was carried out as previously described [21, 26]. Brain homogenate (0.1 mL) was added to the mixture of sodium dodecyl sulphate (0.1 mL, 8.1%), glacial acetic acid (0.75 mL, 20%), thiobarbituric acid (0.75 mL, 0.8%), and 0.3 mL of distilled water. The mixture was heated at 95°C for 1 h in a water bath and pink colour developed indicating presence of MDA. By centrifugation (10,000 r.p.m. for 10 min) supernatant was separated and used for estimation of MDA spectrophotometrically at a wavelength of 532 nm. Simultaneously, protein estimation was performed according to the Lowry method [27]. Finally, MDA content was expressed as *μ*M of MDA per mg of protein for the samples.

#### 2.5.2. Glutathione Estimation

Brain homogenate (0.5 mL) was mixed with sulphosalicylic acid (0.5 mL, 5%) and kept in ice for 30 min for protein precipitation. The supernatant was separated by centrifugation (10,000 r.p.m. for 10 min) at 4°C. Out of it 50 *μ*L of supernatant was taken and mixed with phosphate buffer (450 *μ*L) and 1.5 mL of 5,5′-dithiobis-(2-nitrobenzoic acid) in 0.1 M phosphate buffer (pH 8.0). The mixture was then incubated for 10 min at 37°C. The absorbance was measured at 412 nm spectrophotometrically, using reduced glutathione as an external standard [28, 29].

### 2.6. TNF-*α* and IL-6 Estimation

For determination of TNF-*α* and IL-6 in brain homogenates, standard ELISA protocol was followed (eBioscience ELISA kit). Briefly, cytokine standards and brain homogenates were added to the wells of precoated ELISA plate. The plate was incubated overnight (at 4°C). Then, wells were aspirated and washed 5 times with wash buffer followed by incubation with detection antibody for 1 h at room temperature. Then, after washing, 100 *μ*L Avidin-HRP was added to each well and the plate was incubated for 30 min, followed by washing as per previous steps. Tetramethylbenzidine was added as a substrate followed by 15 min incubation and then reaction was terminated by adding stop solution. The plate was read at 450 nm. Concentrations of TNF*α* and IL-6 were obtained from standard curve and corrected for protein concentrations [30].

### 2.7. DNA Fragmentation Detection

Terminal deoxynucleotidyl transferase mediated dUTP nick end labeling (TUNEL) assay was done to identify the extent of DNA fragmentation. Prior to isolation, in situ fixation of brain was carried out by transcardial perfusion using 4% buffered paraformaldehyde in saline (pH 7.4). Isolated brains were dehydrated and embedded in wax. Serial brain sections of 5 *μ*m thickness were cut using microtome (Leica, Germany) from the mid-brain regions. The 3′ end of the fragmented DNA was labeled using the DNA fragmentation detection kit (Calbiochem, USA) manufacturer following manufacturer instructions. Briefly, the brain sections were prepared for labeling reaction by rehydration followed by treatment with proteinase K. After adding TdT equilibration buffer, the specimens were incubated with labeling reaction mixture in a humidification chamber (90 min, 37°C). The sections were mounted and observed under fluorescent microscope and images were acquired with the CCD camera (Leica, Germany). All the images were taken in a double blind manner. Total cell population and TUNEL positive cells were counted using the image analysis software “Leica Qwin” (Leica, Germany). TUNEL positive cells were expressed as percentage of total cells [31, 32].

### 2.8. Immunohistochemistry

Rats were anaesthetized and transcardially perfused with ice cold phosphate buffered saline (PBS), followed by 4% buffered paraformaldehyde saline (pH 7.4) for in situ fixation of the brain. Brains were isolated, dehydrated, and embedded in paraffin and a series of 5 *μ*m sections were made using microtome (Leica, Germany). After hydration the sections were washed with Tris-buffered saline (TBS, pH 7.4) followed by incubation with proteinase K for 20 minutes for antigen retrieval. After washing with TBS, the sections were incubated with blocking buffer (5% normal goat serum in PBS) for 2 hours. Endogenous biotin binding sites were blocked by sequential incubation of avidin and biotin for 30 minutes each. The sections were then incubated with primary antibody (for tyrosine hydroxylase, Sigma) in blocking buffer at 4°C overnight. The sections were washed three times with PBS and then incubated with biotin-conjugated anti-rabbit IgG (Sigma-Aldrich Inc., USA) in blocking buffer for 2 hours. The specific labelling was detected using diaminobenzidine (DAB). The sections were counterstained with hematoxylin and observed under light microscope (Leica, Germany) and images were acquired with the CCD camera (Leica, Germany) [30]. The entire study was carried out in a double blind manner.

### 2.9. Physiologically Based Pharmacokinetic Simulation of Fenofibrate Plasma Profiles in Rat Model

Fenofibrate is a neutral, lipophilic and BCS class II compound. After oral administration, fenofibrate is converted rapidly to its active metabolite (fenofibric acid) through the hydrolysis of the ester bond by plasma esterase within the gut wall and liver [33]. Thus, the fenofibric acid plasma concentrations versus time profiles were simulated following oral administration of micronize suspension of fenofibrate. ACAT model, integrated in the GastroPlus program (Version 8.5, Simulations Inc.), was used to characterize the kinetics of fenofibric acid in the brain. The ADMET Predictor program (Version 5.5, Simulations Inc.) was used to predict biopharmaceutical properties (e.g., LogP, solubility, permeability, pKa) from structure and subsequently used as input in GastroPlus for simulation of fenofibric acid. The input parameters used during the simulation of plasma concentrations versus time profiles of fenofibric acid are summarized in Table 1.

*In vivo *plasma concentration profiles of fenofibric acid after oral administration of 27 mg/kg micronize suspension in rat were obtained from the literature [33, 34]. As recommended by FDA guidance documents, the similarity factor (*f*_2_) was calculated to compare the observed and simulated profiles. The *f*_2_  value can range from 0 to 100. According to the FDA guide, *f*_2_ value >50 indicates that two profiles are similar to each other with the difference of less than 10%. A model-independent *f*_2_ value was calculated by using the following formula:

(1) $$\begin{array}{c} f_{2}=50\;\text{lo}{\text{g}}_{10}\;\left[\frac{100}{\sqrt{1+\left(1/n\right)\sum_{t=1}^{n}{\left(R_{t}-T_{t}\right)}^{2}}}\right], \end{array}$$

where *R*_*t*_ = relative fraction of observed plasma drug concentration compared to the *C*_max⁡_ value at time *t*, *T*_*t*_ = relative fraction of simulated plasma drug concentration compared to the *C*_max⁡_ value at time *t*, and *n* = number of sampling time points [35].

### 2.10. Statistical Analysis

Results were expressed as Mean ± S.E.M. Sigma Stat 2.0 software was used for statistical analysis. Significance of difference between the two groups was evaluated using Student's *t*-test. For the multiple comparisons one way analysis of variance (ANOVA) was used. If ANOVA showed significant difference, then post hoc analysis was performed with Tukey's test. *P* < 0.05 was considered statistically significant.

## 3. Results and Discussion

### 3.1. Effect of PPAR Agonist on Cognitive Impairment

The passive avoidance task is a fear-aggravated test used to evaluate cognitive functions in rodent models in Parkinson's disease [21]. In our study, acquisition trial (AT) was carried out for this test on D + 17 of the experiment in which initial trial latency (ITL) was determined. It was observed that there was no statistically significant difference between the mean latency times of all the groups (Figure S1 in the Supplementary Material available online at http://dx.doi.org/10.1155/2014/753587).

**Figure 1:**
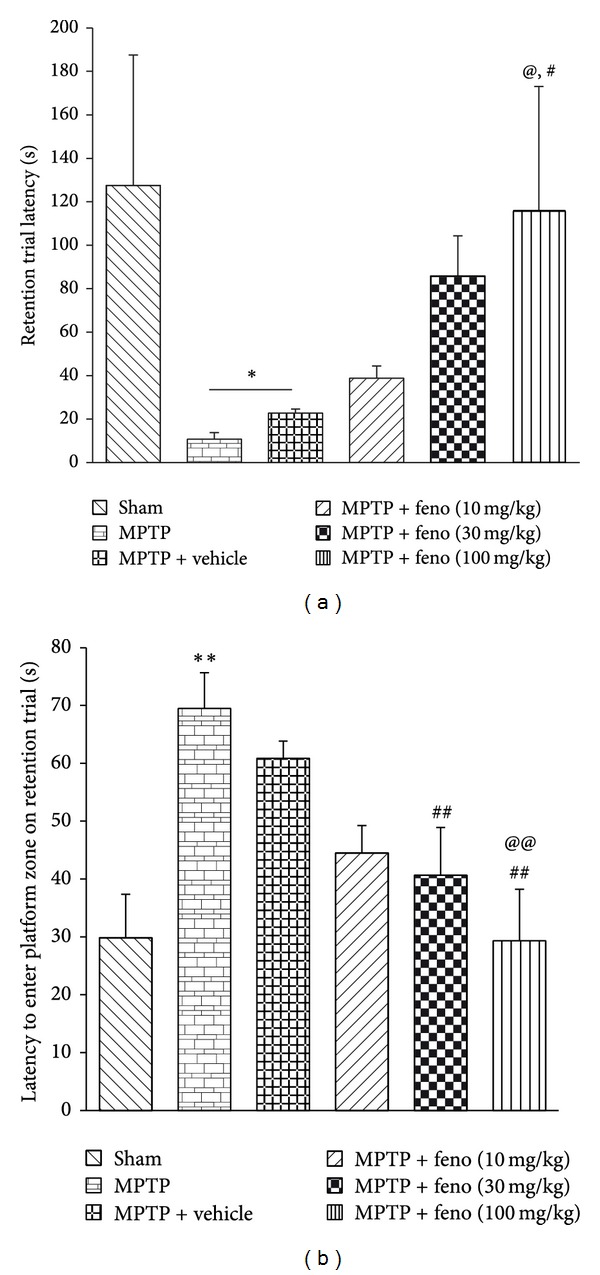
(a) Retention trial latency in passive avoidance test. (b) Latency to enter the platform zone during retention trial in MWM test. *P < 0.05 MPTP or MPTP + vehicle versus sham, **P < 0.01 MPTP versus sham, ^#^ *P* < 0.05 MPTP + vehicle versus MPTP + feno 100 mg/kg treated group, ^##^ *P* < 0.01 MPTP versus feno 30 mg/kg or feno 100 mg/kg treated groups, ^@^ *P* < 0.05 MPTP versus MPTP + fenofibrate 100 mg/kg, ^@@^ *P* < 0.01 MPTP + vehicle versus MPTP + feno 100 mg/kg treated group. All readings are expressed as mean ± SEM (*n* = 8 to 12).

However, during retention trial (RT) which was carried out on D + 18, it was observed that MPTP group had retention trial latency (RTL) significantly lower from the sham group (*P* < 0.01). Similarly, a significant difference in RTL was observed in fenofibrate 10, 30, and 100 mg/kg groups when compared to MPTP treated group (*P* < 0.01). A significant difference was also observed between vehicle treated group with sham group and fenofibrate 100 mg/kg treated group (Figure 1(a)). Neuroprotective potential of PPAR-*α* agonist is substantiated further by a study in which oleoylethanolamide (an endogenous PPAR-*α* agonist) at a dose of 5 mg/kg ameliorated methylenedioxy-methamphetamine induced cognitive deficits in mice [36].

Morris water maze task was used to study spatial learning and memory [37]. In our study, during AT there was a gradual decrease in the latency to reach the platform zone in all groups, but the decrease was significant in fenofibrate treated groups compared to MPTP and vehicle treated groups. Latency in MPTP group was also decreased during five days, but the decrease was much less when compared to the sham operated group. There was a significant difference observed for latency to enter platform zone between MPTP or vehicle treated groups with sham group. Fenofibrate 10, 30, and 100 mg/kg treatment showed a significant decrease when compared to the MPTP and vehicle treated groups (Figure S2).

During RT on the sixth day in MWM test, latency to first entry to the platform zone was significantly lower in sham operated animals, when compared to the MPTP group. There was significant difference observed in the latency in fenofibrate 30 mg/kg (*P* < 0.01) and fenofibrate 100 mg/kg (*P* < 0.01) treated groups, when compared to MPTP and MPTP + vehicle treated groups (Figure 1(b)).

The results indicated that MPTP animals and vehicle treated animals failed or found the platform lately during the RT. Also number of entries into the platform zone was significantly higher in sham and fenofibrate 100 mg/kg treated group (*P* < 0.01) compared to MPTP group (Figure S3). There was no significant difference in the overall average speed observed among different groups (Figure S4). The path was also tracked to observe the behavior of the animals of the different groups (Figure S5). Our result stands in line with other studies where fenofibrate improved cognition in Huntington's disease and cerebral ischemia models [38, 39].

### 3.2. Biochemical Parameters

**Figure 2:**
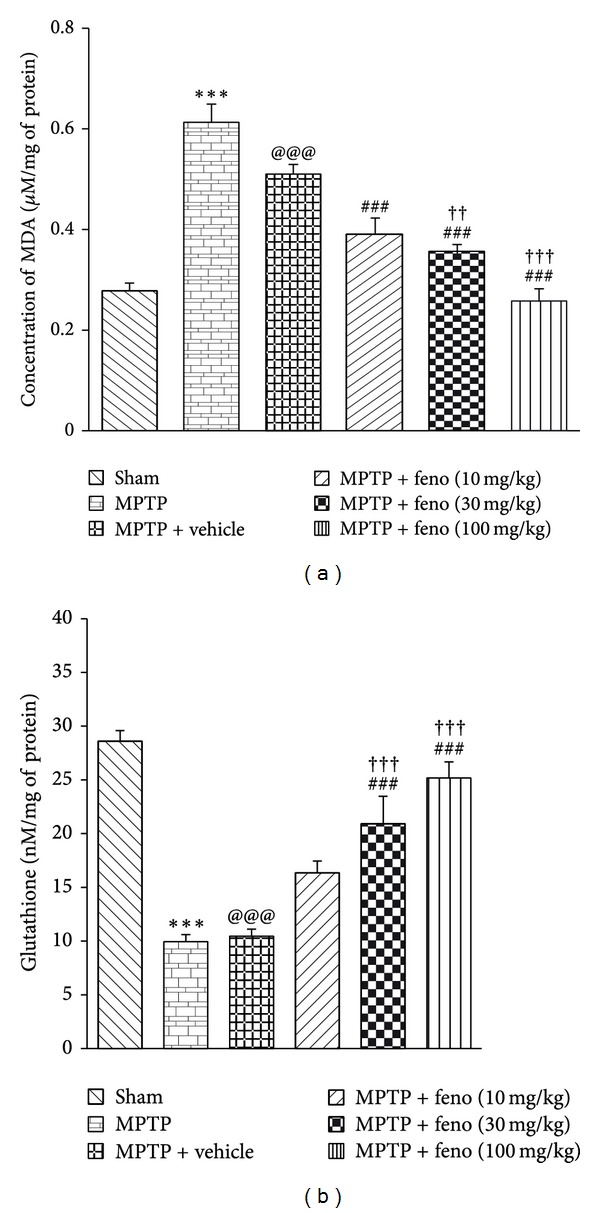
(a) Effect of fenofibrate on MDA and (b) GSH level in different groups. ***P < 0.001 MPTP versus sham, ^###^ *P* < 0.001 MPTP versus feno 10 mg/kg, feno 30 mg/kg and feno 100 mg/kg treated groups, ^@@@^ *P* < 0.001 MPTP + vehicle versus sham and ^†††^ *P* < 0.001 MPTP + vehicle versus feno 100 mg/kg group, ^††^ *P* < 0.01 MPTP + vehicle versus feno 30 mg/kg treated group. All readings are expressed as mean ± SEM (*n* = 8 to 12).

Malondialdehyde is a thiobarbituric acid reactive substance which is a useful parameter for indicating lipid peroxidation in [40]. Intranigral administration of MPTP caused substantial increase in the level of the MDA in the insulted brain when compared to the sham operated group. Fenofibrate at 10, 30, and 100 mg/kg doses significantly decreased the malondialdehyde level (*P* < 0.001). There was also a significant increase of MDA level observed in the vehicle treated group. Fenofibrate at 30 and 100 mg/kg doses significantly decreased MDA level when compared to vehicle treated group (Figure 2(a)).

Reduced glutathione level indicates oxidative stress in the body and estimation is an important parameter in the estimation of oxidative stress [41]. Intranigral administration of MPTP caused substantial decrease in the level of the glutathione in the insulted brain when compared to the sham operated group, but there is a substantial increase in the glutathione in the fenofibrate treated groups when compared to MPTP and vehicle treated groups (*P* < 0.001) (Figure 2(b)).

### 3.3. TNF-*α* and IL-6 Levels

**Figure 3:**
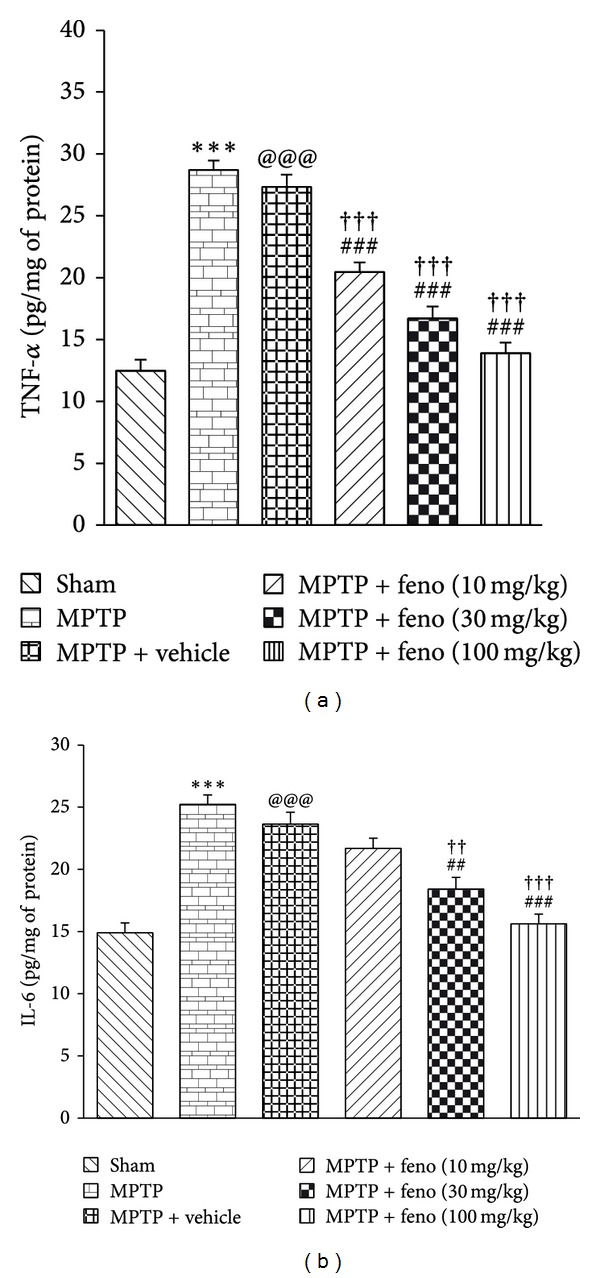
(a) Effect of fenofibrate on TNF-*α*, (b) IL-6 level in different groups. ***P < 0.001 MPTP versus sham, ^@@@^ *P* < 0.001 MPTP + vehicle versus sham; ^##^ *P* < 0.01, ^###^ *P* < 0.001 MPTP versus feno 10, 30, and 100 mg/kg treated groups; and ^††^ *P* < 0.01, ^†††^ *P* < 0.001 MPTP + vehicle versus feno 10, 30, and 100 mg/kg groups. All readings are expressed as mean ± SEM (*n* = 3).

Interleukins and TNF-*α* are the mediators and serve as biological markers in inflammation [42]. In our study, there was an elevation in TNF-*α* and IL-6 levels in the brains of MPTP treated rats as compared to sham group. In fenofibrate treated rats, there was also observed a significant decrease in the levels of TNF-*α* and IL-6 levels (Figure 3). This result substantiated the anti-inflammatory properties of fenofibrate as reported in a previous study [43].

### 3.4. DNA Fragmentation

**Figure 4:**
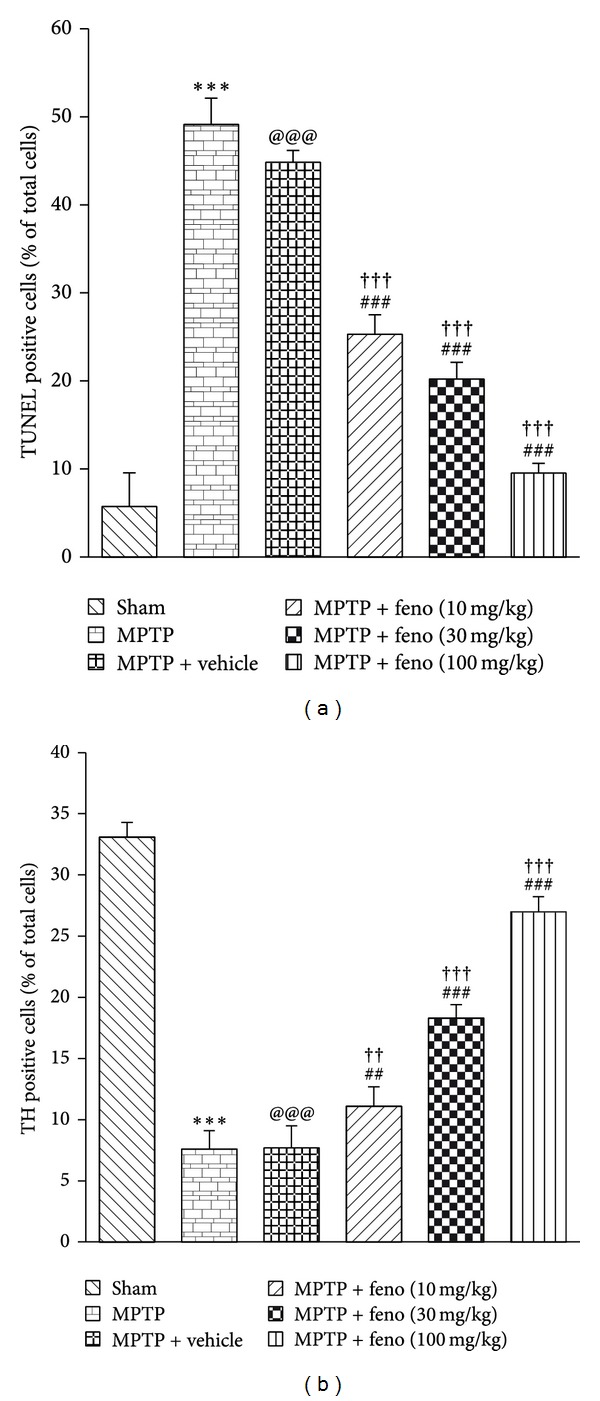
(a) Quantification of DNA fragmentation observed in TUNEL assay. (b) Tyrosine hydroxylase immunohistochemistry and effect of fenofibrate. ***P < 0.001 MPTP versus sham, ^@@@^ *P* < 0.001 MPTP + vehicle versus sham, ^##^ *P* < 0.01, ^###^ *P* < 0.001 MPTP versus feno 10, 30, and 100 mg/kg treated groups, ^††^ *P* < 0.01, ^†††^ *P* < 0.001 MPTP + vehicle versus feno 10, 30, and 100 mg/kg group. All readings are expressed as mean ± SEM (*n* = 3) and images were acquired at 40X magnification.

Delayed neuronal cell death in MPTP brain was postulated in several studies [44]. TUNEL assay was carried out to check the existence of apoptotic DNA fragmentation in PD brain. Brain sections from MPTP treated animals showed a significantly higher percentage of cells with DNA damage as indicated by TUNEL +ve cells when compared with brain sections from sham group. Fenofibrate treatment significantly reduced the number of TUNEL +ve cells when compared to MPTP treated group which was almost similar to sham group (Figure S6 and Figure 4(a)).

### 3.5. Immunohistochemistry

We studied tyrosine hydroxylase (TH) immunolocalization in brain microsections in substantia nigra regions. We found decreased TH immunopositive cells in MPTP treated brains (Figure S7). TH immunopositive cells were found to be more pronounced in fenofibrate treated groups when compared to MPTP treated group and vehicle group (Figure 4(b)). Our result was further supported by a previous report in which fenofibrate treatment ameliorated MPTP-induced degeneration of TH +ve neurons in mice [20].

### 3.6. Simulated Plasma Profiles of Fenofibric Acid Using GastroPlus Software

**Figure 5:**
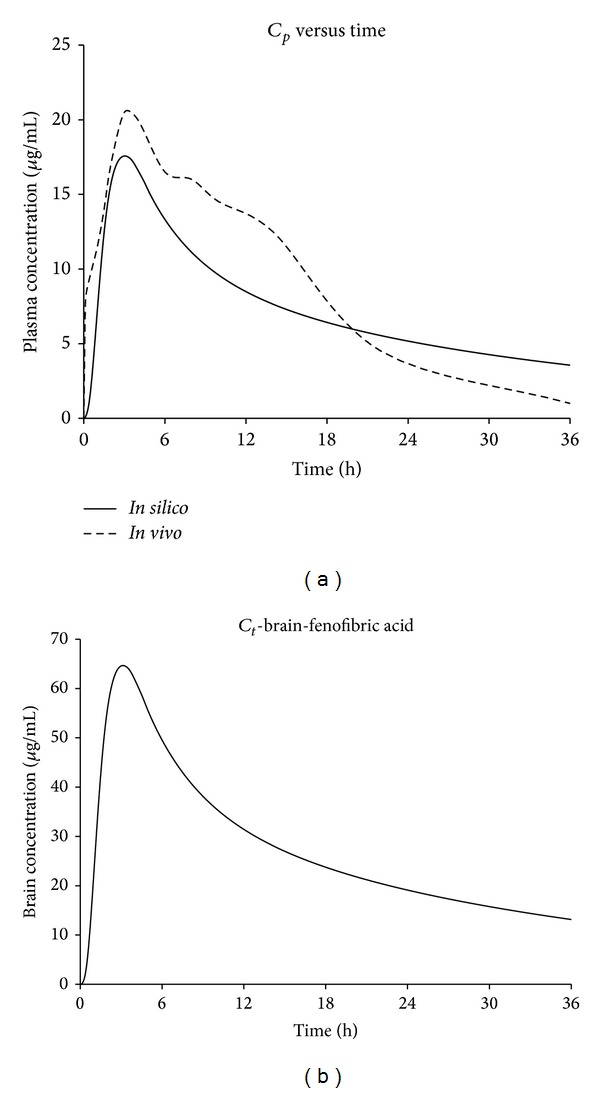
(a) Simulated and observed fenofibric acid plasma concentration profiles for micronize suspension at dose strength of 27 mg/kg in the fasted state. (b) The concentration profile of fenofibric acid in the brain.

The simulated and observed fenofibric acid plasma concentration profiles for the micronize suspension at a dose strength of 27 mg/kg in the fasted physiological state are shown in Figure 5(a).

The simulated and observed pharmacokinetic parameters of fenofibric acid in rat models are summarized in Table 2. The point estimate ratios for AUC_0–*t*_, *C*_max⁡_, and *T*_max⁡_ were found to be 0.99, 0.86, and 0.80, respectively. The comparison of *in vivo* and *in silico* plasma concentration profile gives the *f*_2_ value of 67.37. The concentration profile of fenofibric acid in the brain is shown in Figure 5(b).

In summary, based on above findings, we suggest that PPAR-*α* agonist is neuroprotective in PD-induced cognitive impairments.

## Supplementary Material

Supplementary information provide the results and photomicrographs obtained in Passive Avoidance test, Morris Water Maze test, TUNEL assay and Immunohistochemistry studies.

## Figures and Tables

**Table 1:** The input parameters used during the simulation of plasma concentrations versus time profiles of fenofibric acid in rat model.

Sr. number	Input data	Value
1	MW	262.5
2	log⁡*P*	4.01
3	p*K*_*a*_ (acidic)	3.56
4	Dose	27 mg/kg
5	Lower limit reference solubility (pH 7.4)	0.0294 mg/mL
6	Diffusion coefficient	0.825 × 10^−5^ cm^2^/s
7	Particle density	1.2 g/mL
8	Effective particle radius	5 µm
9	Effective permeability	7.66 × 10^−4^ cm/s
10	Physiology	Fasted conditions
11	Absorption model	Opt logD Model SA/V
12	Stomach transit time	0.1 h
13	Body weight	0.25 kg (Rat)
14	Clearance	1.1 mL/min/kg
15	Simulation time	36 h

**Table 2:** The comparison of predicted and *in vivo* pharmacokinetic parameters of fenofibric acid.

Sr. number	Pharmacokinetic parameters	*In vivo *	Predicted	Predicted/*in* *vivo* ratio
1	AUC_0–*t*_ (µg h/mL)	274.68 ± 38.11	271.89	0.99
2	*C*_max⁡_ (µg/mL)	20.49 ± 2.32	17.58	0.86
3	*T*_max⁡_ (hours)	3.77 ± 0.64	3	0.80
4	*f*_2_ Value	67.37		
