# Supplementary material for: Neuroprotective Potential of Peroxisome Proliferator Activated Receptor-α Agonist in Cognitive Impairment in Parkinson's Disease: Behavioral, Biochemical, and PBPK Profile
Source: PPAR Res. 2014 Feb 19;2014:753587. doi: 10.1155/2014/753587 (PMC3945208; doi:10.1155/2014/753587)
Supplement: Supplementary file 1 — Supplementary information provide the results and photomicrographs obtained in Passive Avoidance test, Morris Water Maze test, TUNEL assay and Immunohistochemistry studies. [file 753587.f1.pdf]

FIGURE S1: Initial Trial Latency for different groups in passive avoidance test

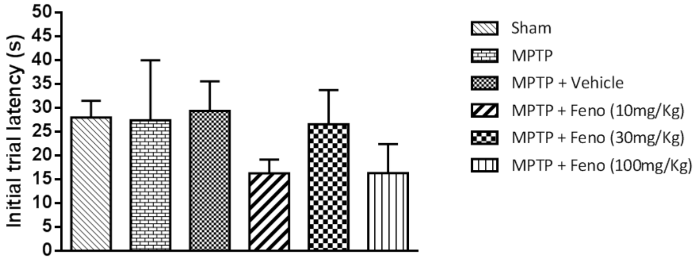

FIGURE S1: Initial Trial Latency for different groups in passive avoidance test. No significant different difference was found among different groups in Initial Trial Latency (n=6 to 10).

FIGURE S2: Latency to first entry to the platform zone for different groups during AT in MWM test

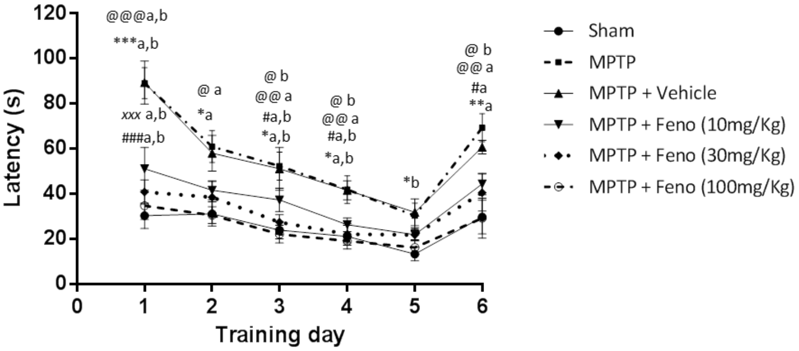

FIGURE S2: Latency to first entry to the platform zone for different groups. *** $p < 0.001$, ** $p < 0.01$, * $p < 0.05$ (a) MPTP vs sham, (b) MPTP + Vehicle vs sham; ^{xxx} $p < 0.001$ (a) MPTP vs fenofibrate 10 mg/kg, (b) MPTP + Vehicle vs fenofibrate 10 mg/kg; $^{\#\#\#}$ $p < 0.001$, $^{\#\#}$ $p < 0.01$, $^{\#}$ $p < 0.05$ (a) MPTP vs fenofibrate 30 mg/kg, (b) MPTP + Vehicle vs fenofibrate 30 mg/kg, $^{@@@}$ $p < 0.001$, $^{@@}$ $p < 0.01$, $^{@}$ $p < 0.05$ (a) MPTP vs fenofibrate 100 mg/kg, (b) MPTP + Vehicle vs fenofibrate 100 mg/kg. All readings are expressed as mean ± SEM (n=8 to 12).

FIGURE S3: Number of entries to the platform zone for different groups during RT in MWM test

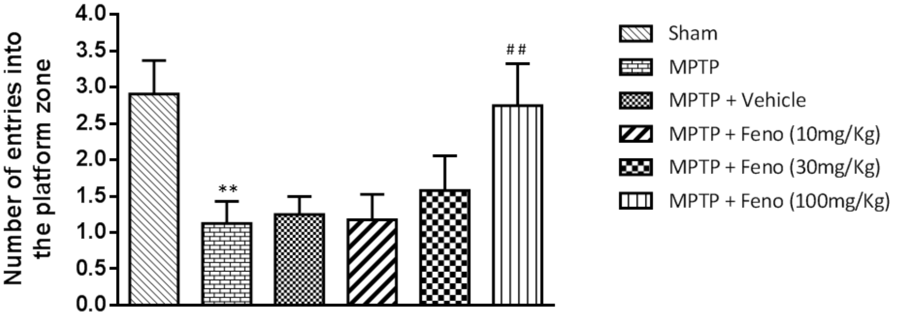

FIGURE S3: Number of entries to the platform zone for different groups during retention trial. ** $p < 0.01$ MPTP vs sham, $^{\#\#}p \leq 0.01$ MPTP vs MPTP + fenofibrate 100 mg/kg group. All readings are expressed as mean ± SEM (n=8 to 12).

FIGURE S4: Overall average speed for different groups on day 5 in Morris Water Maze test

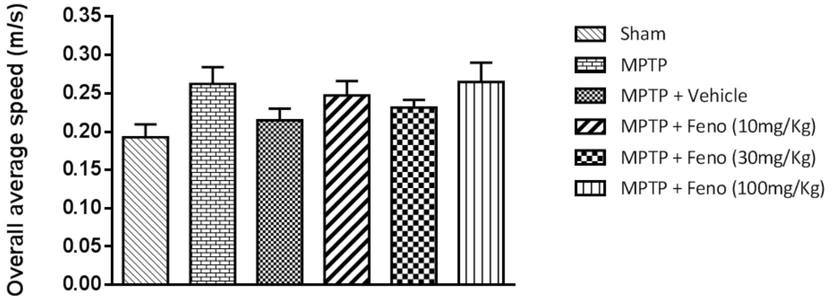

FIGURE S4: Overall average speed for different groups on day 5 in Morris Water Maze test. No significant different difference was found among different groups in overall average speed. All readings are expressed as mean ± SEM (n=8 to 12).

FIGURE S5: Representative track-plots on Day 5 in MWM test

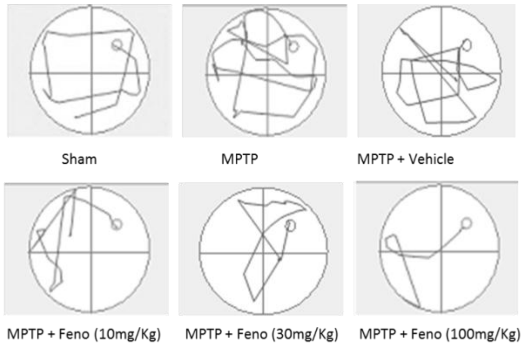

FIGURE S5: Representative path tracked for sham group, MPTP group and fenofibrate treated groups during day 5, indicating the latency to entry into the platform zone.

FIGURE S6: Representative microphotographs in TUNEL assay

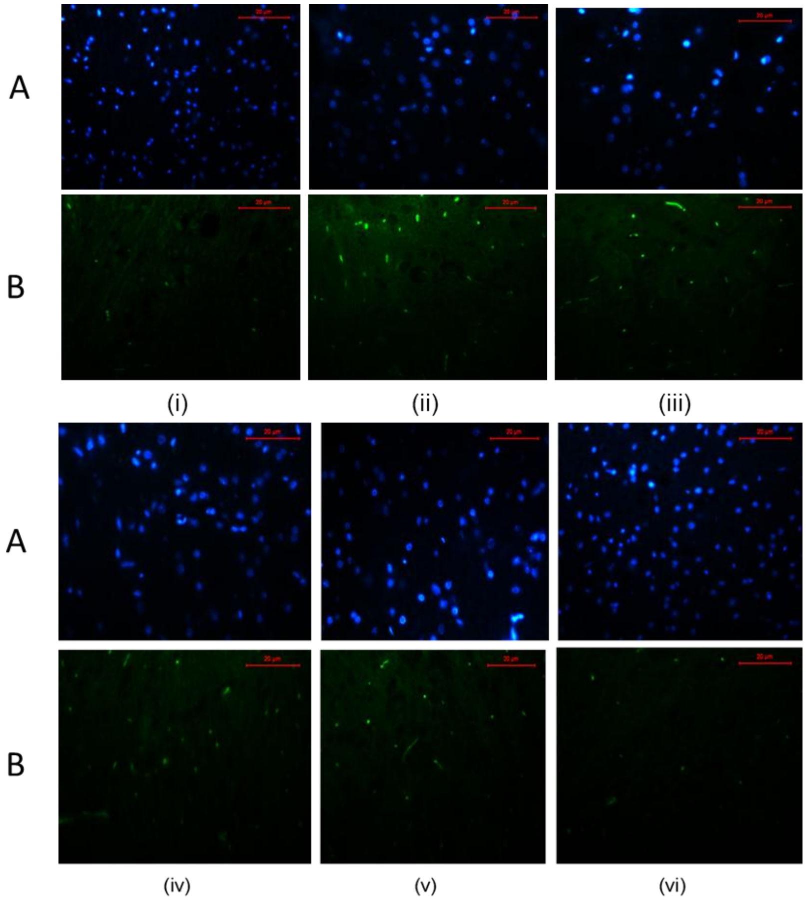

FIGURE S6: Representative microphotographs in TUNEL assay showing (A) DAPI stained total cells (B) TUNEL positive cells in substantia nigra region; (i) Sham, (ii) MPTP, (iii) MPTP + vehicle (iv) MPTP + Feno 10 mg/kg (iv) MPTP + Feno 30 mg/kg, (vi) MPTP + Feno 100 mg/kg

FIGURE S7: TH immunohistochemistry to estimate TH immunopositive cells

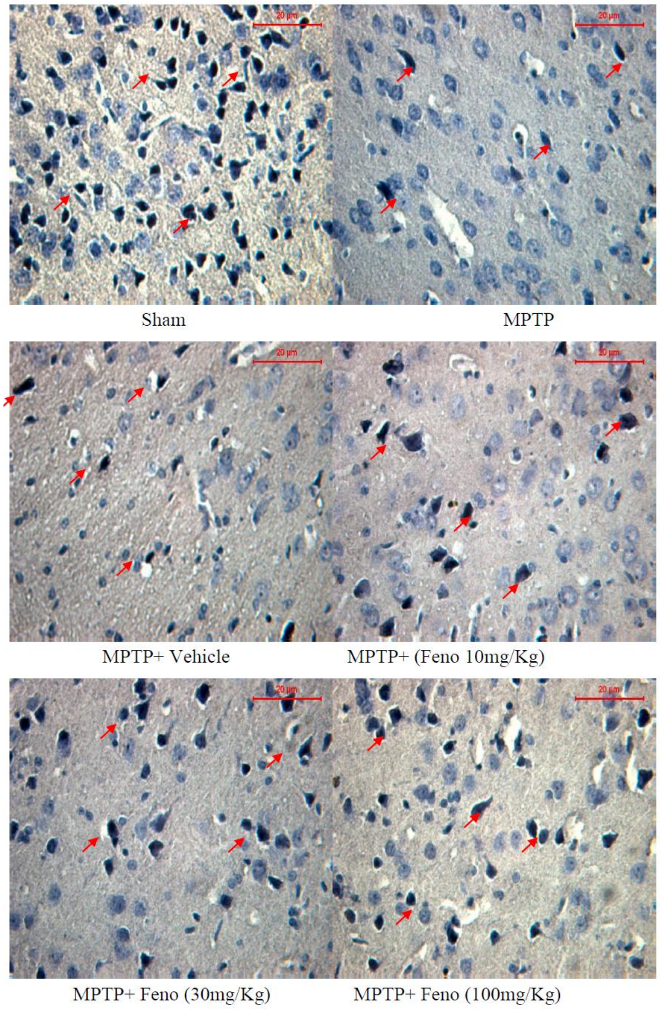

FIGURE S7: Images of immunohistochemistry to estimate TH immunopositive cells (indicated by red arrows) in substntia nigra regions. All the images were acquired at 40X magnification.
